# Hospital-based antimicrobial stewardship, India

**DOI:** 10.2471/BLT.22.288797

**Published:** 2022-11-09

**Authors:** Sonam Vijay, V Ramasubramanian, Nitin Bansal, VC Ohri, Kamini Walia

**Affiliations:** a Division of Epidemiology and Communicable Diseases, Indian Council of Medical Research, New Delhi, Ansarinagar, 110029 India.; b Department of Infectious Diseases, Apollo Hospitals, Chennai, India.; c Division of Infectious Diseases, Rajiv Gandhi Cancer Institute and Research Centre, New Delhi, India.

## Abstract

**Objective:**

To establish a framework for implementing antimicrobial stewardship in Indian tertiary care hospitals, and identify challenges and enablers for implementation.

**Methods:**

Over 2018–2021 the Indian Council of Medical Research followed a systematic approach to establish a framework for implementation of antimicrobial stewardship in Indian hospitals. We selected 20 Indian tertiary care hospitals to study the feasibility of implementing a stewardship programme. Based on a questionnaire to lead physicians before and after the intervention, we assessed progress using a set of process and outcome indicators. In a qualitative survey we identified enablers and barriers to implementation of antimicrobial stewardship.

**Findings:**

We found an improvement in various antimicrobial stewardship implementation indicators in the hospitals after the intervention. All 20 hospitals conducted monthly point prevalence analysis of cultures compared with three hospitals before the intervention. The number of hospitals that initiated formulary restrictions increased from two to 12 hospitals and the number of hospitals that started practising prescription audit and feedback increased from six to 16 hospitals. Respondents in 15 hospitals expressed their willingness to expand the coverage of antimicrobial stewardship implementation to other wards and intensive care units. Six hospitals were willing to recruit the permanent staff needed for antimicrobial stewardship activities.

**Conclusion:**

Antimicrobial stewardship can be implemented in Indian tertiary hospitals with reasonable success, subject to institutional support, availability of trained manpower and willingness of hospitals to support antimicrobial stewardship-related educational and training activities.

## Résumé

**Objectif:**

Établir un cadre pour la mise en œuvre d'une gestion des antimicrobiens dans les hôpitaux de soins tertiaires en Inde, et identifier les défis et les moteurs de cette mise en œuvre.

**Méthodes:**

Durant la période comprise entre 2018 et 2021, le Conseil indien de la recherche médicale a adopté une approche systématique visant à instaurer un cadre pour assurer la gestion des antimicrobiens dans les hôpitaux indiens. Nous avons sélectionné 20 hôpitaux de soins tertiaires en Inde afin d'étudier la faisabilité du déploiement d'un tel programme. En nous fondant sur un questionnaire soumis aux médecins-chefs avant et après l'intervention, nous avons évalué les progrès au moyen d'une série d'indicateurs relatifs aux processus et aux résultats. Par le biais d'une enquête qualitative, nous avons déterminé quels étaient les obstacles et facteurs favorables à la mise en œuvre d'une gestion des antimicrobiens.

**Résultats:**

Nous avons constaté une amélioration au niveau de plusieurs indicateurs de mise en œuvre dans les hôpitaux à l'issue de l'intervention. Les 20 établissements ont tous mené chaque mois une analyse de prévalence ponctuelle des cultures, alors qu'ils n'étaient que trois à le faire avant l'intervention. Le nombre d'hôpitaux ayant introduit une dispensation contrôlée est passé de deux à 12, et ils sont désormais 16 à vérifier et réguler les prescriptions, contre six auparavant. Dans 15 établissements, les participants ont exprimé leur volonté d'étendre ces pratiques à d'autres services et unités de soins intensifs. Enfin, six hôpitaux étaient prêts à recruter le personnel requis pour accomplir des tâches liées à la gestion des antimicrobiens.

**Conclusion:**

La mise en œuvre de ce type de gestion peut rencontrer un certain succès dans les hôpitaux tertiaires en Inde. Ce succès dépend notamment de l'aide qu'apportent les institutions, de l'accès à une main-d'œuvre qualifiée et de la volonté des hôpitaux à soutenir les activités de formation et d'éducation à la gestion des antimicrobiens.

## Resumen

**Objetivo:**

Establecer un marco para la aplicación del programa de optimización del uso de antimicrobianos en los hospitales de atención especializada de la India, e identificar los desafíos y los factores que facilitan la aplicación.

**Métodos:**

Entre 2018 y 2021, el Consejo Indio de Investigación Médica siguió un enfoque sistemático para establecer un marco que permitiera la aplicación de la optimización del uso de antimicrobianos en los hospitales de la India. Se seleccionaron 20 hospitales de atención especializada de la India para estudiar la viabilidad de implementar un programa de optimización de uso. A partir de un cuestionario dirigido a los médicos principales antes y después de la intervención, se evaluaron los avances mediante un conjunto de indicadores de procesos y desenlaces. En una encuesta cualitativa, se identificaron los factores que facilitan la optimización del uso de antimicrobianos y los obstáculos que la dificultan.

**Resultados:**

Se encontró una mejora en varios indicadores de optimización del uso de antimicrobianos en los hospitales después de la intervención. Los 20 hospitales realizaron análisis mensuales de prevalencia puntual de cultivos, en comparación con tres hospitales antes de la intervención. La cantidad de hospitales que iniciaron restricciones en el formulario aumentó de dos a 12 hospitales y la cantidad de hospitales que comenzaron a practicar la auditoría de prescripción y la retroalimentación aumentó de seis a 16 hospitales. Los encuestados de 15 hospitales expresaron su voluntad de ampliar la cobertura del programa de optimización del uso de antimicrobianos a otras salas y unidades de cuidados intensivos. Seis hospitales estaban dispuestos a contratar el personal permanente necesario para las actividades de optimización del uso de antimicrobianos.

**Conclusión:**

La optimización del uso de antimicrobianos se puede aplicar en los hospitales de atención especializada de la India sin problemas, siempre que se cuente con el apoyo de las instituciones, la disponibilidad de personal capacitado y la voluntad de los hospitales de apoyar las actividades educativas y de formación relacionadas con la optimización del uso de antimicrobianos.

## ملخص

الغرض وضع إطار عمل لتنفيذ الإشراف على مضادات الميكروبات في مستشفيات الرعاية من الدرجة الثالثة الهندية، وتحديد التحديات والعوامل المساعدة على التنفيذ.

الطريقة خلال الفترة من 2018 إلى 2021، اتبع المجلس الهندي للأبحاث الطبية أسلوبًا منهجيًا لإنشاء إطار عمل لتنفيذ الإشراف على مضادات الميكروبات في المستشفيات الهندية. قمنا باختيار 20 مستشفى رعاية من الدرجة الثالثة لدراسة جدوى تنفيذ برنامج الإشراف. بناءً على استطلاع رأي للأطباء الرئيسيين قبل التدخل وبعده، قمنا بتقييم التقدم باستخدام مجموعة من مؤشرات العملية والنتائج. في مسح نوعي، قمنا بتحديد العوامل المساعدة على تنفيذ الإشراف على مضادات الميكروبات، والمعوقات التي تحول دون تنفيذ هذا الإشراف.

النتائج لاحظنا تحسنًا في المؤشرات المختلفة لتنفيذ الإشراف على مضادات الميكروبات في المستشفيات بعد التدخل. أجرت جميع المستشفيات الـ 20 تحليلًا شهريًا لانتشار النقاط للثقافات مقارنة بثلاثة مستشفيات قبل التدخل. زاد عدد المستشفيات التي بدأت قيود التركيبات من مستشفيين إلى 12 مستشفى، وبدأ عدد المستشفيات التي تمارس التدقيق في الوصفات الطبية والمراجعة من ستة إلى 16 مستشفى. أعرب المشاركون في 15 مستشفى عن رغبتهم في توسيع تغطية تنفيذ الإشراف على مضادات الميكروبات، لتشمل الأجنحة ووحدات العناية المركزة الأخرى. كانت ستة مستشفيات ترغب في تعيين الموظفين الدائمين اللازمين لأنشطة الإشراف على مضادات الميكروبات.

الاستنتاج يمكن تنفيذ الإشراف على مضادات الميكروبات في المستشفيات الهندية من الدرجة الثالثة مع إحراز نجاح معقول، في ظل وجود الدعم المؤسسي، وتوافر القوى العاملة المدربة، ورغبة المستشفيات في دعم الأنشطة التعليمية والتدريبية المتعلقة بالإشراف على مضادات الميكروبات.

## 摘要

**目的:**

旨在建立印度三级保健医院实施抗菌药物管理的框架，并确定实施管理所面临的挑战和推动因素。

**方法:**

在 2018 至 2021 年期间，印度医学研究理事会采用系统方法建立了印度医院实施抗菌药物管理的框架。我们选择了 20 家印度三级护理医院，对实施管理计划的可行性进行研究。根据一项就干预前后情况对指导医师开展的问卷调查，我们使用一组过程和结果指标对进展进行了评估。在一项定性调查中，我们确定了实施抗菌药物管理的推动因素和障碍。

**结果:**

我们发现，在实施干预后医院各项抗菌药物管理实施指标均有改善。所有 20 家医院均进行了月度文化流行率分析，而仅有三家医院在实施干预之前开展了该项分析。实施处方限制的医院数量从 2 家增加到 12 家，开始实施处方审核和反馈的医院数量从 6 家增加到 16 家。15 家医院的受访者表示，他们愿意将抗菌药物管理实施的覆盖面扩大到其他病房和重症监护病房。6 家医院愿意招聘长期工作人员，负责抗菌药物管理工作。

**结论:**

在印度三级医院实施抗菌药物管理工作可取得一定的成功，这取决于机构支持、可用的接受过培训的人力资源以及医院支持与抗菌药物管理相关的教育和培训活动的意愿。

## Резюме

**Цель:**

Создать основу для реализации стратегии использования противомикробных средств в больницах третьего звена в Индии, а также определить комплексы задач и факторы, способствующие ее реализации.

**Методы:**

В течение 2018–2021 гг. Индийский совет по медицинским исследованиям придерживался системного подхода к созданию основы для реализации стратегии использования противомикробных средств в больницах Индии. Для изучения возможности реализации программы управления лекарственными средствами было выбрано 20 больниц Индии, относящихся к третьему звену. На основе опросника для ведущих врачей до вмешательства и после него была проведена оценка прогресса с помощью набора показателей процесса и результатов. В ходе качественного исследования авторы определили факторы, способствующие и препятствующие реализации стратегии использования противомикробных средств.

**Результаты:**

Авторы отметили улучшение различных показателей реализации стратегии использования противомикробных средств в больницах после вмешательства. Во всех 20 больницах ежемесячно проводился точечный анализ распространенности культур по сравнению с показателями трех больниц до вмешательства. При этом количество больниц, которые начали вводить ограничения доступа к отдельным антибиотикам, увеличилось с двух до двенадцати, а количество больниц, которые начали практиковать аудит рецептов и обратную связь, увеличилось с шести до шестнадцати. Респонденты из 15 больниц выразили готовность расширить охват стратегии использования противомикробных средств на другие отделения и блоки интенсивной терапии. В шести больницах выразили готовность нанять постоянный персонал, необходимый для проведения мероприятий по использованию противомикробных средств.

**Вывод:**

Стратегия использования противомикробных средств может быть достаточно успешно реализована в больницах третьего звена в Индии при условии оказания организационной поддержки, наличия подготовленных кадров и готовности больниц поддерживать образовательные и учебные мероприятия, связанные с применением противомикробных средств.

## Introduction

In recent years, several low- and middle-income countries, including India, have reported alarming rates of antimicrobial resistance. The *Global action plan on antimicrobial resistance* released by the World Health Organization (WHO) recognized effective implementation of hospital antimicrobial stewardship programmes as one of the key priorities to protect the efficacy of antimicrobials.^1^^,^^2^Antimicrobial stewardship is a coordinated set of cost-effective interventions which support the use of microbiological data to rationalize antimicrobial prescriptions and reduce their adverse effects.^3^ India’s national action plan for antimicrobial resistance (2017–2021) also recognized antimicrobial stewardship as a key strategy to optimize and reduce the use of antimicrobial drugs.^4^ However, India does not yet have a national-level antimicrobial stewardship plan.

There are several barriers to implementing an effective antimicrobial stewardship programme in low- and middle-income countries.^5^^,^^6^ The foremost barrier is that guidance for establishing such programmes has primarily been developed for hospitals in high-income countries and is often unsuitable for the health-care systems in low- and middle-income countries. The implementation of antimicrobial stewardship in high-income settings is driven by infectious disease physicians and pharmacists, and in some hospitals the entire antimicrobial stewardship programme is pharmacist-led.^7^ A lack of trained manpower, especially infectious disease physicians and pharmacists, makes it challenging to establish antimicrobial stewardship in low- and middle-income countries such as India, especially in public sector hospitals.^8^

The Indian Council of Medical Research undertook a survey in 2015 to understand the capacity available in Indian hospitals for implementing antimicrobial stewardship programmes. The survey highlighted many gaps and challenges. Since then, the council has taken several steps to address these gaps.^9^ As a preparatory step, in 2017 the council organized capacity-building workshops for hospital teams to sensitize them to the core principles of antimicrobial stewardship.^9^ Here we report the systematic approach followed by the council to implement antimicrobial stewardship interventions in Indian tertiary hospitals.^10^ We describe our experience of creating the framework for the interventions – the processes followed, challenges faced and progress made – and suggest mechanisms to sustain antimicrobial stewardship in low- and middle-income settings.

## Methods

### Setting

In 2017 the Indian Council of Medical Research established a network of tertiary care hospitals to participate in antimicrobial resistance surveillance.^10^ We conducted this implementation study in all 20 hospitals of the network, which cover a wide range of geographical regions of India. The selected hospitals included 14 government and six private hospitals. All hospitals provided services to both urban and rural populations. The authorized bed capacity of these 20 hospitals ranged from 373–4500 (intensive care unit 18–360 beds) with an average bed occupancy of 75–100%. The study was approved by the institutional human ethics committees of the participating hospitals.

### Intervention

The first step in the series of initiatives in 2017 was to put together a generic protocol and seek local hospital commitment and support. We held a meeting with hospital administrators to discuss the skilled manpower and logistic requirements needed to implement the protocol. After that, hospitals were invited to participate in an antimicrobial stewardship study. An introductory workshop was held for participants in 2018. The discussions following the workshop helped participants to understand the feasibility of initiating an antimicrobial stewardship programme in their hospital. Participants identified easily attainable objectives which could be translated into achievable results, while deferring consideration of more challenging objectives.^11^

**Fig. 1:**
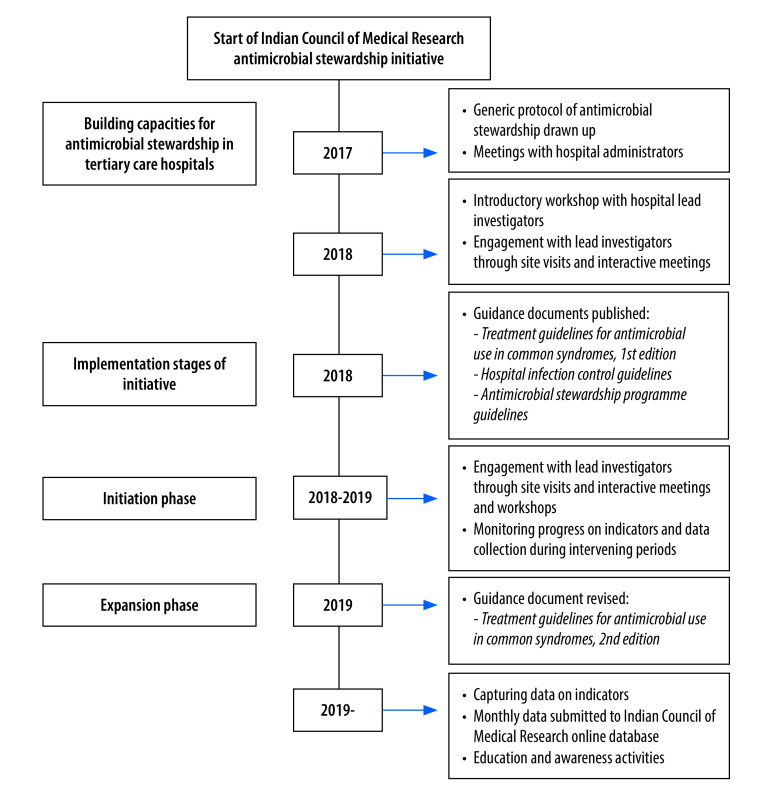
Timeline of the Antimicrobial Stewardship Programme Implementation and Research Initiative of the Indian Council of Medical Research

In 2018 we conducted a cross-sectional study to collect information on antimicrobial stewardship policies and practices being followed in the participating hospitals. We recruited a clinician or intensive care physician from each hospital to act as lead investigator. Lead physicians answered an email questionnaire on the antimicrobial stewardship practices in their hospital (available in data repository)^12^. The questions included whether the institution had a written antimicrobial stewardship document and stewardship committee; whether, and how often, they monitored microbial resistance data and antibiotic prescribing practices; and whether they had a hospital antibiogram and formulary restrictions. Based on the insights from the baseline assessment, we devised a stepwise approach for implementation of an antimicrobial stewardship programme in Indian tertiary hospitals (Fig. 1).

Using information from the baseline assessment and consultations, we determined a key set of activities for the participating hospitals to undertake in the next 3-year period (Box 1). These activities were the foundation of the council’s Antimicrobial Stewardship Programme Implementation and Research Initiative (ASPIRE) and formed the basis of a set of process and outcome indicators for monitoring the programme.

Box 1 **Key activities and indicators for assessing the implementation of an antimicrobial stewardship programme in Indian hospitals**

**Initiation phase (2019): process indicators**

- Set up a hospital antimicrobial stewardship committee.
- Create an antibiotic policy based on the hospital antibiogram.
- Undertake point prevalence surveys of antimicrobial resistance from cultures.
- Record antibiotic consumption in intensive care units (days of therapy and defined daily doses).
- Initiate prescription audits for carbapenems and polymyxin prescriptions in intensive care units.
- Initiate formulary restrictions.
- Implement initial or minimal level of de-escalation of antibiotic use.^a^
- Organize awareness and education workshops for staff on antimicrobial stewardship.

**Expansion phase (2020): process and outcome indicators**

- Expand the implementation of antimicrobial stewardship within hospital to increase the coverage to10% of total beds (10% of intensive care beds and 10% of non-intensive care beds).
- Monitor adherence to hospital antibiotic policies.
- Continue capturing antibiotic consumption and prescription audits.
- Classify antibiotic consumption data as per the AWaRe classification of the WHO: Access, Watch or Reserve.^13^
- Record patients’ clinical outcomes: cured and discharged; left hospital against medical advice; or died.

WHO: World Health Organization.^a^ De-escalation criteria were: stopping antibiotics within 5 days; changing from combination to monotherapy; and changing narrower spectrum intravenous drugs to oral formulations.

### Implementation

The antimicrobial stewardship interventions were implemented in the 20 participating hospitals over 2019–2021; most hospitals were able to initiate activities in the first quarter of 2019. We provided guidance to all participating hospitals through periodic meetings and workshops conducted by the council. To implement the programme, the council provided funding of 15 000 United States dollars annually to each hospital for up to 3 years to cover expenses for skilled manpower (two new positions, a pharmacist and a nurse, in each hospital), non-recurring expenditure (such as computers), consumables and funding for trainings, meetings, transportation and other costs.

### Assessment

We monitored progress and evaluated the implementation of the stewardship programme in its initiation and expansion phases using the process and outcome indicators summarized in Box 1.^14^ In the initiation phase, the study was limited to selected beds in intensive care units. In the expansion phase we had proposed to expand the coverage of antimicrobial stewardship to 10% of total beds. 

To assess the progress made, lead physicians from the participating hospitals repeated the questionnaire from the baseline assessment in 2019. We also performed a comparative analysis of data collected from hospitals before and after the intervention. We drew up a set of process and outcome indicators to evaluate. All the hospitals entered data using the online platform developed by the council.^12^ Antibiotic consumption was measured using days of therapy and defined daily doses as units. Key activities undertaken in the first year were: (i) point prevalence analysis of antimicrobial resistance of cultures; (ii) initiation of formulary restrictions; (iii) prescription audits; (iv) constitution of antimicrobial stewardship committees; and (v) creation of antibiogram-based guidelines. In the expansion phase, in addition to the activities in the initiation phase, hospitals analysed: (i) antibiotic prescriptions; (ii) adherence to hospital policies; and (iii) clinical outcomes. Antibiotics prescribed to patients were analysed according to the World Health Organization’s Access, Watch, Reserve (AWaRe) classification. Clinical outcomes from intensive care units and ward patients’ data were recorded as cured and discharged; left against medical advice; or death. Each hospital conducted the point prevalence survey of cultures every month of the study period and recorded rates of de-escalation of antibiotic use. 

We also did a qualitative survey to obtain feedback on the hospitals’ experience of the programme via an email questionnaire (available in the data repository).^12^ Lead physicians were asked about their views on antimicrobial stewardship for health-care institutions; their motivation for working on the programme; challenges faced during the implementation of antimicrobial stewardship in their hospital and their suggested solutions; and positive changes after implementation of antimicrobial stewardship.

Individual hospitals did their own data analysis and uploaded antimicrobial consumption data to the online data system. The data was received and analysed at the Indian Council of Medical Research coordination unit from all the participating hospitals in the form of annual reports. 

## Results

### Assessment of progress

#### Multidisciplinary team

**Fig. 2:**
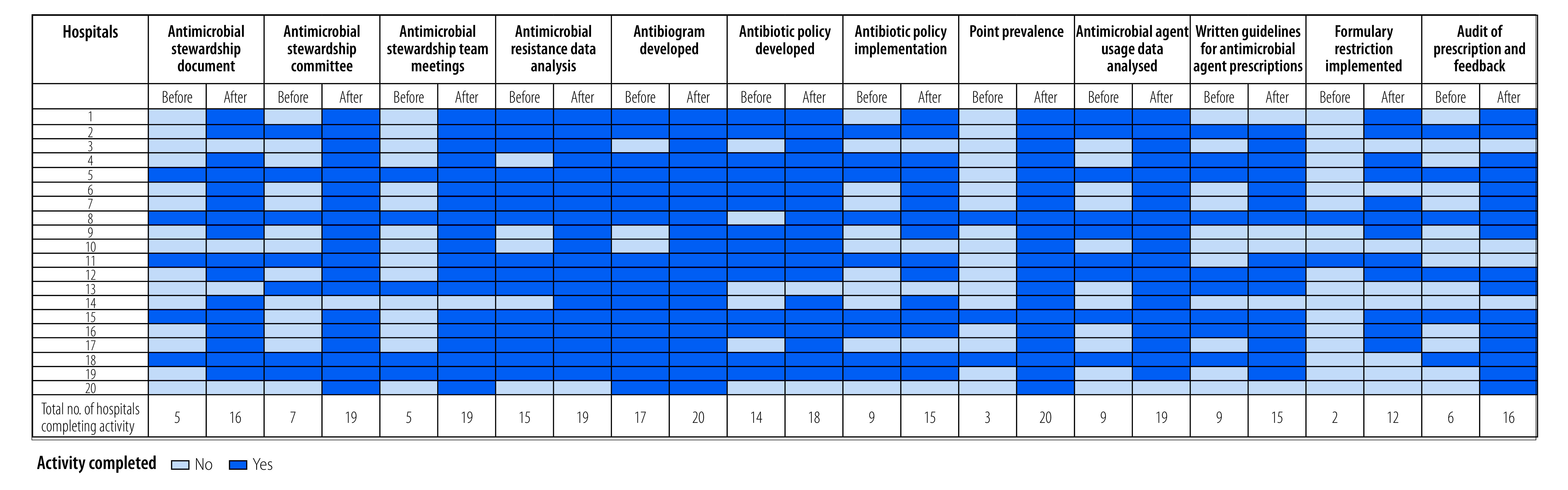
Activities completed before and after implementation of the antimicrobial stewardship intervention in 20 Indian tertiary care hospitals, 2019–2021

Note: We analysed data from before the intervention in February 2018 and after the intervention in June 2019.

The improvements in the various antimicrobial stewardship parameters before and after the intervention in hospitals are summarized in Fig. 2. The data show that after the intervention, 19 of the 20 hospitals (95%) had established antimicrobial stewardship teams as compared with only seven hospitals (35%) before the intervention. After the intervention, a physician, clinical microbiologist and hospital administrator were part of the antimicrobial stewardship committee in 19 hospitals (95%), a clinical pharmacist in 17 hospitals (85%), a nurse in 12 hospitals (60%) and an infectious disease specialist in seven hospitals (35%). Nineteen hospitals (95%) conducted regular antimicrobial stewardship team meetings after the intervention, of which nine hospitals (45%) organized quarterly meetings and five hospitals (25%) half-yearly meetings. These data demonstrate substantial improvements as only five hospitals (25%) had these meetings before the intervention. Full details of the progress in antimicrobial stewardship after the intervention are shown in the data repository.^12^

#### Guidelines and policies

After the intervention, all 20 hospitals were able to create the hospital antibiogram and 18 hospitals were able to develop an antibiotic policy based on the antibiogram. Capacity-building workshops on antimicrobial stewardship were organized by all hospitals, compared with only one hospital before implementation of the intervention. Overall, the 20 hospitals conducted training for 2377 medical and paramedical staff over the study period, thus expanding the provision of antimicrobial stewardship education after the intervention.

#### Improvement strategies

Analyses of antimicrobial resistance and monitoring of antimicrobial use were performed by 19 hospitals (95%) compared with only 15 hospitals (75%) before the intervention period. The practice of formulary restriction was introduced by 12 hospitals (60%), of which six hospitals reported restrictions on the use of colistin, four hospitals restricted polymyxin B, four hospitals restricted ceftazidime–avibactam and tigecycline, three hospitals restricted carbapenems and two hospitals restricted linezolid and fosfomycin. Only two hospitals (10%) reported having formulary restrictions before the intervention period. After the intervention 16 hospitals (80%) introduced prescription audit and feedback, compared with only six (30%) hospitals that used it before the intervention. A point prevalence survey of cultures from patients before initiation or change in antibiotics was carried out by all 20 hospitals (100%) after the intervention, compared with only three hospitals before the intervention (15%).

**Fig. 3:**
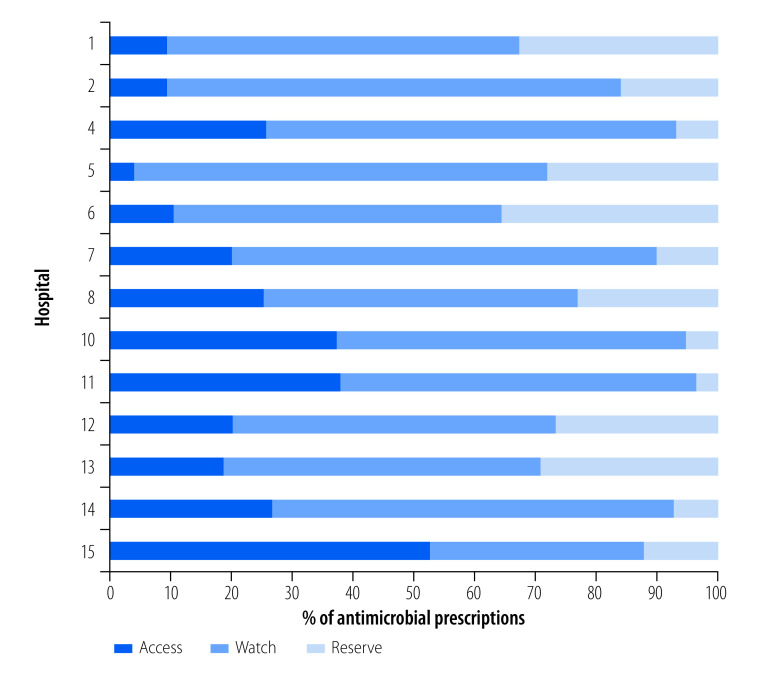
AWaRe classification of 14 168 antibiotic prescriptions in 13 Indian tertiary care hospitals, 2019–2021

Note: The bars show the percentage of total antibiotics prescribed in each hospital, classified according to the World Health Organization Access, Watch, Reserve (AWaRe) groups.^13^

Fifteen hospitals were able to obtain clinical outcome data from a total of 20 691 patients in the expansion phase of the initiative. The full data are shown in the data repository.^12^ Fig. 3 (available at: https://www.who.int/publications/journals/bulletin/) shows the AWaRe groups of 14 168 antibiotic prescriptions in 13 of these hospitals. The proportion of antibiotics prescribed from the Access group ranged from 4% to 52% across the hospitals. We found that across all hospitals, 2752 (19%) antibiotics were prescribed from the Access group, 8732 (62%) antibiotics were prescribed from the Watch group and 2684 (19%) antibiotics were prescribed from the Reserve group.

### Participants’ experiences

According to the qualitative survey, 12 hospitals (60%) had a positive experience of the antimicrobial stewardship implementation project according to the lead physician. Respondents reported that activities undertaken in the study were helpful in creating awareness about the appropriate choice of antimicrobials and the correct dosage and duration of use among the medical staff of their hospital. Antimicrobial stewardship committees in 19 hospitals (95%) conducted regular meetings and undertook annual updates of the antibiotic policy. Nine hospitals (45%) reported improvement in obtaining cultures before the use of antimicrobial drugs or any change in antimicrobials. This approach was helpful in restricting unnecessary antibacterial and antifungal use as reported by eight hospitals (40%). Respondents in all hospitals expressed their desire and willingness to continue antimicrobial stewardship as a permanent activity, while six hospitals (30%) have also assigned permanent hospital staff for management of antimicrobial stewardship after completion of the project. Overall, physicians in 16 hospitals (80%) saw value in expanding the coverage of antimicrobial stewardship to more intensive care units than those included in the current study. Antimicrobial stewardship committees in 14 hospitals (70%) have decided to continue the stewardship activities after the completion of the project, including measuring antimicrobial consumption by prescription audit in intensive care units, carrying out monthly point prevalence studies, implementing formulary restrictions and conducting educational and awareness activities for staff on antimicrobial stewardship.

## Discussion

The core elements for setting up effective antimicrobial stewardship in hospitals requires a structure and resources that are usually not available in hospitals in low- and middle-income countries.^11^ The Indian Council of Medical Research supported 20 Indian tertiary care hospitals to set up a framework for implementing antimicrobial stewardship by providing funding and the necessary training and guidance. Our findings document that nearly all hospitals that participated in the study appreciated the importance of implementation of antimicrobial stewardship strategies and welcomed the initiative. 

The available guidelines on antimicrobial stewardship from high-income countries clearly specify that infectious disease specialists and clinical pharmacists are the pillars of hospital antimicrobial stewardship.^15^^,^^16^ Indian hospitals have a shortage of both professions, as highlighted in our previous survey.^8^ In the current study, we addressed this challenge by choosing intensive care physicians and clinicians (paediatricians and surgeons) to lead this initiative in their respective hospitals. These professions are likely to be the most committed to antimicrobial stewardship and all the participants led the projects with good success at all hospitals, irrespective of their specialization.

Our study showed that 19 out of 20 hospitals were able to formulate a multidisciplinary team and undertake capacity-building activities that led to successful implementation of the proposed activities. Capacity-building is one of the key components for instituting antimicrobial stewardship programmes in low- and middle-income countries.^17^ Furthermore, providing clinical pharmacists to hospitals led to recognition of the value a clinical pharmacist brings to the quality of antimicrobial prescribing. In our study, antimicrobial stewardship driven by clinical pharmacists led to 80% of hospitals practising audit and feedback and 60% of hospitals applying formulary restrictions on the use of many broad-spectrum antimicrobials compared with 30% and 10% of hospitals, respectively, before the intervention.

All 20 hospitals were able to measure the process indicators during the first year of the study but, due to the coronavirus disease 2019 (COVID-19) pandemic, data on the outcome indicators were available in only 15 hospitals. All hospitals conducted point prevalence surveys of antimicrobial resistance in cultures after the antimicrobial stewardship intervention. All hospitals performed appropriate antimicrobial sensitivity tests from bacterial or fungal cultures for all patients who were prescribed parenteral antimicrobials. In our study, prescriptions of antibiotics from the Access group were in the range of 4–52%, contrary to the WHO AWaRe policy-based indicator which recommended that more than 60% of antibiotics prescribed should be from the Access group.^18^ A 2019 study of antibiotic sales data from India also documented minimum consumption of antibiotics from the Access group.^19^ Antimicrobial stewardship activities suffered a setback in all hospitals due to the COVID-19 pandemic response. Reluctance to accept formulary restrictions and de-escalation of therapy in some hospitals was also among the key challenges that we identified.

Through this study we wanted to evaluate whether hospitals were receptive to changes after antimicrobial stewardship interventions. Overall, the hospital administrators and clinicians were supportive of introducing antimicrobial stewardship and the clinicians reported having positive experiences in their institutions. This finding could be attributed to the sensitization meetings with hospital administrators, which the council organized before launching the project. Antimicrobial stewardship implementation in these hospitals demonstrated small but effective changes. 

It is encouraging to note that most of the hospitals were able to initiate the activities to measure the process and outcome indicators, although no trend could be documented. However, the data on outcome indicators continue to be monitored and further findings will reveal trends over time. As a next step, to further enhance the hospital’s antimicrobial stewardship plan for accurate and consistent data collection of antimicrobial utilization, the council has approved funds to these hospitals for collecting data on outcome measures related to antimicrobial stewardship for another 3 years. The next phase will be to understand the impact of the intervention on optimizing antimicrobial selection (drug, dose and duration) and on reducing adverse drug events (morbidity and mortality, length of hospital stay and health-care expenditure). For this phase, hospitals will measure the impact of stewardship on performance indicators such as adherence to guidelines, multidrug resistance rates, clinical outcomes and antimicrobial consumption.^20^ Capturing these outcomes is important not only for evaluating the success of the antimicrobial stewardship at an individual hospital level but also for identifying areas for further improvement.^21^^,^^22^ Although the council is providing funds to these hospitals for a finite period, the hospitals have been asked to identify resources to sustain the antimicrobial stewardship activities beyond completion of the project.

There were many administrative challenges in running this project. One challenge was that senior clinicians and surgeons have little time to spare for the stewardship activities owing to busy clinics and hospital administration responsibilities. Another issue was that principal investigators of the projects, who were mostly clinicians, were not accustomed to managing research grants, and had difficulty finding time from their clinical work. Many sites reported staff leaving in the middle of the project, which also led to interruptions in data collection. Unfortunately, the ongoing antimicrobial stewardship activities were de-prioritized in five hospitals out of 20 (three government and two private hospitals) at the time of the COVID-19 pandemic. During the pandemic, these hospitals faced challenges in accessing intensive care units and gathering information, as most of their intensive care units were converted to COVID care, and because fewer non-COVID patients were admitted during that period.

Nevertheless, our experience shows that – in the absence of infectious disease specialists – intensive care physicians or other clinicians can lead effective antimicrobial stewardship activities in hospitals. Administrative support for antimicrobial stewardship within the hospital and the availability of clinical pharmacists exclusively for antimicrobial stewardship, supported through hospital funds, would be crucial for sustaining this activity in Indian hospitals.

With the increasing levels of microbial resistance to all the broad-spectrum antimicrobials in India,^23^ there is an urgent need to prioritize antimicrobial stewardship. Optimizing and reducing the use of antimicrobials (strategic priority No. 4 of India’s national action plan) needs to be achieved through implementation of mandatory national antimicrobial stewardship at least in all tertiary and secondary level hospitals. The tertiary care hospitals included in this study offer the advantage of having multidisciplinary teams, which are crucial for implementation of antimicrobial stewardship. From our experience, we envision that implementing an effective antimicrobial stewardship programme in secondary hospitals is going to be even more challenging, in terms of infrastructure constraints, financial constraints, lack of trained manpower and lack of higher-level support. Besides that, microbiology laboratories in low-resource settings do not have quality control systems, infrastructure and trained manpower.^24^ Factors which are key for the successful implementation of antimicrobial stewardship programmes in low- and middle-income countries include fostering the political willpower, involvement of clinical leadership, organizational commitment and creation of mandatory national guidelines for implementation of antimicrobial stewardship. Recently, the Indian National Medical Commission has made it mandatory for all medical colleges to have a functional antimicrobial stewardship committee.^25^ These developments are encouraging and, if implemented effectively, would be valuable for establishing antimicrobial stewardship as a permanent activity in medical colleges. The challenge will be to hold the attention of policy-makers and health administrators to prioritize antimicrobial stewardship so that the resources necessary for its implementation are made available across the health-care systems.

Our study has some limitations. First, we were not able to study the clinical and microbiological impact of the antimicrobial stewardship programme in any of these hospitals due to the short duration of the study and to the COVID-19 pandemic disrupting the implementation. Second, this study was focused on creating a basic framework for antimicrobial stewardship in hospitals and piloting a set of interventions that can be applied to Indian hospitals; we did not assess the quality of its implementation. Finally, we were not able to document any change in prescription patterns (such as de-escalation rates) as per the AWaRe classification. We believe that establishing an antimicrobial stewardship framework in any hospital is the first step towards the larger goal of rational antimicrobial prescribing. Despite these limitations, we believe that our study highlights the key steps which are needed to implement antimicrobial stewardship in low- and middle-income countries.

In conclusion, most hospitals implemented antimicrobial stewardship programmes in their hospitals with the help of funding and capacity-building activities from the Indian Council of Medical Research. Administrative support and the availability of clinical pharmacists supported through hospital funds would be crucial for sustaining antimicrobial stewardship activity. Our study highlights the importance of developing the capacity of multidisciplinary teams, identifying local problems and finding innovative local solutions. 
